# Un nuevo giro a los estudios sobre ciencia y religión: la cuestión del nacionalismo

**DOI:** 10.1590/S0104-59702024000100070

**Published:** 2024-12-16

**Authors:** Miguel de Asúa

**Affiliations:** i Academia Nacional de la Historia. Ciudad de Buenos Aires – Argentina mdeasua@yahoo.com

Desde su surgimiento en la década de 1980 (hay antecedentes pioneros como el de Richard S. Westfall), los estudios sobre ciencia y religión han crecido hasta constituirse en un área muy vital dentro de la historiografía de la ciencia. Como bien se destaca en la introducción al volumen que reseñamos, gran parte de esta producción deriva, inmediata o de manera remota, de la “tesis de Merton”. En la línea historiográfica en habla inglesa, que parte del libro canónico de John Brooke (1991), continúa en el intento de extender el área a otras religiones que la cristiana (Brooke, Numbers, 2011) y culmina en la discusión que puso en primer plano la historicidad de la noción de “ciencia” tanto como de la de “religión” (Harrison, 2015), hay una secuencia lógica que, en el fondo de los fondos, permanece dentro de los marcos culturales del mundo anglo-protestante. Con nuestro estudio sobre las relaciones entre ciencia, religión y secularización en Argentina (Asúa, 2023) presentamos un caso de larga duración en una sociedad con el tipo de secularización de las sociedades originalmente católicas, con lo que se buscó desencajar estos estudios de sus limitaciones de origen y ubicarlos en un marco de mayor interés para el mundo iberoamericano. El nuevo libro editado por Jaume Navarro y Kostas Tampakis es una contribución muy interesante al área: plantea la ambiciosa (y novedosa) pregunta por las relaciones entre ciencia, religión y nacionalismo y lo hace a través de contribuciones de autores que constituyen una muestra equilibrada, con mayoría de sociedades “occidentales” no-angloparlantes (hay dos latinoamericanos). En la introducción, los editores discuten tres vastos cuerpos historiográficos: (a) el tema de las naciones, los nacionalismos y las identidades nacionales; (b) ciencias y religiones; y (c) ciencias y nacionalismos. Sobre la base de la tesis de la “ciencia como nación” (con referencia a David Cahan), argumentan que la construcción de las narrativas que buscaron apuntalar esta mítica comunidad de la ciencia (una “nación” muy internacional, por cierto) habrían utilizado la “tesis del conflicto” como manera de legitimarse a sí mismas a través de la diferenciación con otras actividades humanas (en este caso, la de las instituciones que median lo sagrado): “Así – dicen los editores – la persistencia de la tesis del conflicto y otros mitos sobre ciencia y religión pueden ser vistos como la consecuencia natural del intento de modelar la noción de ciencia en contraste o en oposición a otras prácticas epistémicas, morales o políticas” (Navarro, Tampakis, 2024, p.13).

Seguimos la propia categorización de los editores en la recapitulación de las diferentes contribuciones. Un primer grupo de artículos analiza el empleo de figuras científicas con valencia religiosa por discursos nacionalistas. M. Alper Yalçinkaya considera los intentos de destacar el elemento específico “turco” dentro del campo de las “contribuciones islámicas a la ciencia” en los años posteriores a la creación de la República de Turquía (1923), con concentración en Ibn Sina (Avicena, 980-1037). Neil Tarrant analiza el énfasis sobre la orientación científica de *La città dei sole* de Tommaso Campanella (1602), en la historiografía de corte nacionalista-liberal del historiador Luigi Firpo (1915-1989), en los años posteriores a la Segunda Guerra Mundial, como parte de la interpretación de este historiador sobre las condiciones intelectuales en la Contra-reforma. Un segundo grupo de contribuciones tiene que ver con el papel del nacionalismo en las polémicas anti-cristianas o anti-católicas. Como continuación de sus estudios previos, James C. Ungureanu analiza la importancia del énfasis nacionalista en la producción histórica de John W. Draper previa a su *A history of the conflict between science and religion* (1874) en el contexto de los enfrentamientos resultados de la inmigración católica a EEUU; Draper (que era inglés) habría percibido, dice el autor, que este tipo de inmigrante constituía un desafío para la edificación de una sociedad estadounidense abierta y tolerante (“nativismo americano”). Jeffrey T. Zalar estudia la importancia de las ciencias naturales en las relaciones inter-confesionales en la Alemania de Bismarck, con acento en las intersecciones entre católicos y ciencia en el contexto de la nacionalidad (los vínculos entre *Christentum*, *Wissenschaft* y *Vaterland*). El tercer grupo de estudios tiene que ver con la educación. Jaume Navarro discute como los ingenieros en España entre los siglos XIX y XX, en un ambiente intelectual de regeneracionismo y de “la polémica de la ciencia española”, adoptaban posiciones pragmáticas alejadas del conflicto entre el discurso clerical y el anti-clerical y en consonancia con las competencias de su profesión, en función de la contribución de ésta al avance del país. Ignacio Silva se ocupa de las polémicas entre positivistas y católicos en el contexto de la legislación educativa de corte secular en la Argentina de los años 1880, con particular énfasis en los programas educativos y textos de dos positivistas, el naturalista ítalo-argentino Pietro (Pedro) Scalabrini, con base en la ciudad de Paraná, y su discípulo Alfredo Ferreira, influyente educador que se acercó a la “religión de la humanidad” de Comte. El positivismo, de tanto peso en Iberoamérica, es también el tema del trabajo de Juan Manuel Rodríguez Caso, quien estudia dicha corriente de pensamiento y las relaciones entre ciencia y religión en el contexto del nacionalismo mexicano, con acento en autores mexicanos que combinaron agendas nacionalistas con discusiones de ciencia y religión. Kosta Tampakis considera los papeles del profesor universitario y el teólogo universitario (en el modelo alemán) en el ambiente de helenismo nacionalista posterior al reconocimiento de Grecia como Estado en 1828 y muestra como tanto los teólogos ortodoxos griegos como los científicos usaban el ideal irredentista para consolidar sus respectivas agendas. El cuarto grupo de ensayos explora las relaciones entre identidades nacionales incoadas y ciencia. Michał Wagner estudia cómo en el siglo XIX en Polonia, entonces una nación “virtual” partida entre Rusia, Alemania y Austria, fue recibido y decodificado el darwinismo en términos de dos proyectos nacionales para la futura independencia: el de un Estado secular o el de una nación liderada por la nobleza y el clero que defendería valores católicos. El trabajo de Agustín Ceba Herrero y Joan March Noguera considera al sacerdote mallorquín Antoni Maria Alcover y la creación del primer diccionario sistemático de idioma catalán; el estudio científico de la lengua, en la perspectiva de este lingüista, debía servir para cristalizar una identidad nacional y a la vez preservar una moral y una religión. El último artículo de esta colección, debido a Katarzyna Jarosz, estudia los museos soviéticos entre 1917 y fines de la década de 1980 como lugares de apuntalamiento de una narrativa nacional, organizada alrededor del “ateísmo científico” y las variantes que ésta fue adoptando, desde la revolución bolchevique hasta la *Perestroika*.

El conjunto es una muy rica colección de estudios de caso (con desniveles de originalidad y pertinencia que no perjudican la buena calidad del conjunto) que inaugura una novedosa y potencialmente muy fructífera área de investigación, de particular interés para la audiencia latinoamericana, como es la cuestión de las relaciones entre ciencia, religión y nacionalismo. Queda como ejercicio para el/la lector/a articular la discusión de los contenidos de los trabajos compilados con la desafiante tesis propuesta por los editores en su introducción. Celebramos la aparición de este valioso y sugerente libro y esperamos que tenga la amplia y bienvenida recepción que merece.
